# The benefits of ashwagandha (*Withania somnifera*) supplements on brain function and sports performance

**DOI:** 10.3389/fnut.2024.1439294

**Published:** 2024-08-02

**Authors:** Shiyi Guo, Mohammad J. Rezaei

**Affiliations:** ^1^College of Physical Education, LiaoNing Petrochemical University, Fushun, Liaoning, China; ^2^School of Medicine, Tehran University of Medical Sciences, Tehran, Iran

**Keywords:** ashwagandha, sports performance, brain function, antioxidant response, exercise, inflammation

## Abstract

Ashwagandha or Withania somnifera is an herbal plant belonging to the Solanaceae family. Because of its wide range of phytochemicals, ashwagandha root extract has been used in numerous research studies, either alone or in conjunction with other natural plants, for various biomedical applications, which include its anti-microbial, anti-inflammatory, anti-stress, anti-tumor, cardioprotective, and neuroprotective properties. Additionally, it improves endothelial function, lowers reactive oxygen species, controls apoptosis, and improves mitochondrial function. These properties make it a useful treatment for a variety of conditions, including age-related symptoms, anxiety, neurodegenerative diseases, diabetes, stress, arthritis, fatigue, and cognitive/memory impairment. Despite the numerous benefits of ashwagandha supplementation, there have been just four meta-analyses on the herb’s effectiveness in treating anxiety, neurobehavioral disorders, impotence, and infertility. Moreover, no reviews exist that examine how ashwagandha affects antioxidant response and physical sports performance. Consequently, the goal of this study was to analyze the scientific literature regarding the effects of ashwagandha consumption on antioxidant response and athletic performance.

## Introduction

A resistance training program consists of exercises that force skeletal muscles to contract against external resistance. Such regimens frequently cause the body to increase muscle mass and produce greater strength (1, 2). However, studies have shown that prolonged and/or high-intensity exercise can damage muscle tissue. In addition, oxidative stress and inflammatory cytokines damage biomolecules, which may lead to additional muscle injury caused by the rise in free radical levels following muscle damage (3). Physical fitness affects the formation of reactive oxygen species (ROS) and causes changes in antioxidant enzymes, which can lead to different degrees of oxidative damage and lipid peroxidation (4, 5). Fatigue and compromised cellular and muscular function have been linked to oxidative stress (6, 7). Superoxide dismutase (SOD), glutathione peroxidase (GPx), and catalase (CAT) are antioxidant enzymes acting as the first line of defense to prevent the generation of free radicals and ROS (8).

Numerous investigations have evaluated the efficacy of dietary alterations and micronutrient supplements in mitigating the oxidative stress generated by exercise (9, 10). Certain medicinal herbs can improve exercise capacity and prevent illness because they contain antioxidants and antifatigue compounds (11). It was reported that supplementation with ashwagandha may enhance the adaptations and gains produced by exercise, making it a helpful addition to a resistance training regimen. There are some justifications for this theory. Ashwagandha has been shown in studies to enhance muscular strength and coordination as well as cardiorespiratory endurance in healthy, normal individual (12, 13). Ashwagandha is classified as a “rasayana” or Ayurvedic rejuvenation therapy which has been used for ages to promote health, increase lifespan, slow down aging, revitalize the body, and produce overall wellbeing (14, 15). Ashwagandha has been reported to possess an extensive spectrum of pharmacological activity, including analgesic, anti-inflammatory, sedative, hypotensive, anxiolytic, immunomodulatory, central nervous system, cardiac, anabolic, and antioxidant properties (14, 16–18). Moreover, it increases thyroid activity and respiratory function, and relaxes smooth muscle (19). Human studies showed that ashwagandha was well tolerated and linked to an increase in testosterone (20, 21) and a decrease in cortisol (22). Ashwagandha may lessen the rise in blood urea nitrogen, lactic acid, and corticosterone that occurs during stress and exercise (14). It may also lessen the activation of dopamine receptors in the brain during stressful conditions (16, 23).

The fact that ashwagandha has several active ingredients may explain the different mechanisms of action. These include compounds like anaferine, isopelletierine, steroidal lactones (withaferins and withanolides), and saponins (23–25).

As exercise may be thought of as a kind of acute stress, and the stress response after consumption of ashwagandha results in improvements in human physical performance, the adaptagenic properties of ashwagandha means it could act as an active ergogenic supplement. Therefore, we summarize the studies that have investigated the role of ashwagandha in exercise performance, antioxidant responses, and increased adaptation.

## Ashwagandha and antioxidant responses

**Figure 1:**
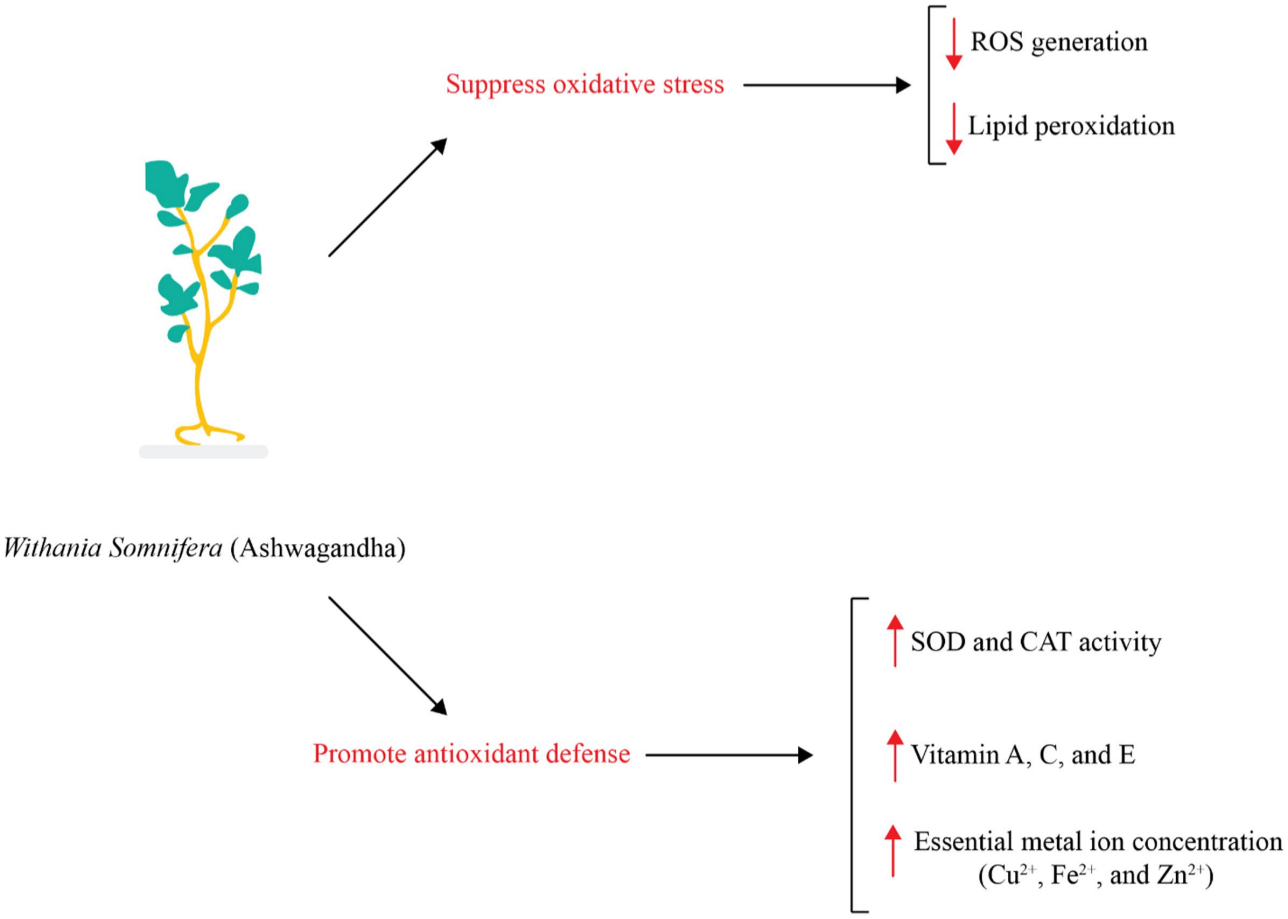
The effects of ashwagandha on antioxidant responses.

**Figure 2:**
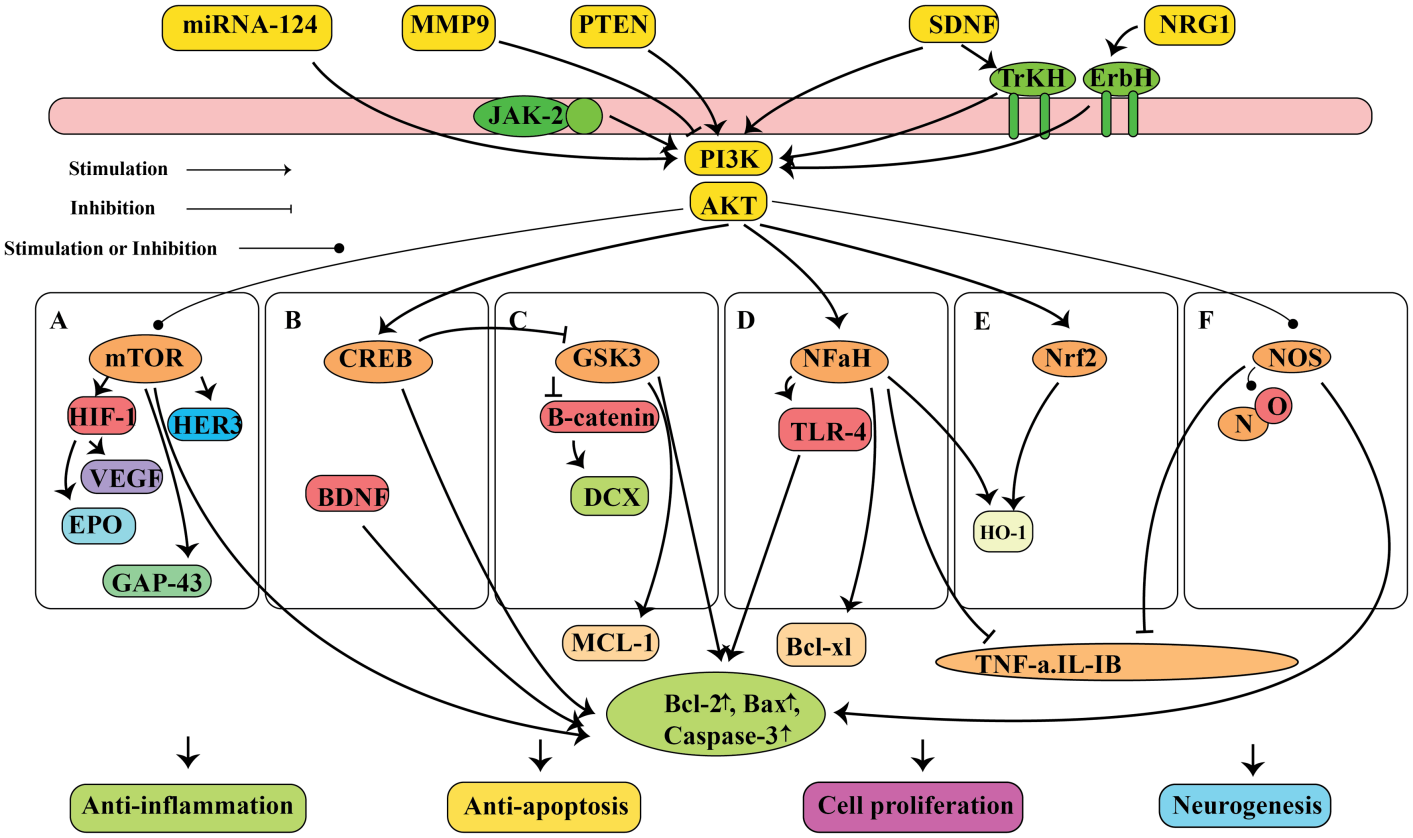
The neuroprotective properties of ashwagandha is attributed to the molecular and pathway mechanisms, such as suppression of inflammation, inhibition of cell death, promotion of cell growth, and stimulation of neurogenesis. These effects are mediated by the PI3K/AKT signaling pathway, which activates different pathways including mTOR, CREB, GSK3, NF-κB, Nrf2, and NOS. This figure adapted from Gu et al. (32).

The antioxidant properties of ashwagandha have been documented using *in vitro* cell culture, *in vivo* animal research, and clinical trials involving healthy individual (24, 26–28). Additionally, researchers have proposed that ashwagandha could be beneficial for those with oxidative stress-related diseases (29). Ashwagandha has been shown to contain high levels of flavonoids, phenolic compounds, and antioxidant compounds (30). Therefore ashwagandha can repair oxidative damage in cells and lipid peroxidation, as well as combat the formation of reactive oxygen species (ROS) as shown in Figure 1. When ROS attack cell membranes, they produce harmful lipid peroxides and malondialdehyde (MDA) (31). Nuclear factor erythroid 2-related factor 2 (Nrf2) controls the gene expression of antioxidant enzymes involved in combating oxidative damage by activating the PI3K/PTEN/Akt pathway. The compound Withaferin A has been found to have beneficial effects on the nervous system, such as preventing cell death and promoting cell growth. This is achieved by inhibiting the protein PTEN and activating the PI3K/AKT/mTOR and PI3K/AKT/GSK3β pathways, as well as preventing the movement of vascular smooth muscle cells (Figure 2) (32, 33). Nrf2 is also strongly induced by withaferin A where it has a cytoprotective effect (34, 35). Antioxidant proteins, including heme oxygenase-1 (HO-1) and heat shock protein 70 (HSP70), as well as antioxidant enzymes like CAT and SOD, glutathione (GSH), GSH reductase, GPx, thioredoxin (Trx), and Trx reductase are all downstream products of Nrf2 activation (36). Since these enzymatic and protein antioxidants do not need to be continuously produced, they show a longer duration of action compared to vitamins and coenzymes, which are depleted during the initial redox reactions (37). In healthy people, the antioxidant function of the bioactive compounds in ashwagandha, support the herb’s usage in promoting oxidative equilibrium and possibly preventing disorders linked to oxidative stress.

## Ashwagandha and brain function

Extensive research has been conducted on the diverse physiological effects of *Withania somnifera* (WS), with a particular focus on its potential use to treat brain disorders (14, 38). The effects of WS root and WS leaf on the nervous system have been investigated in both preclinical and clinical studies. Two recent reviews have comprehensively compiled and analyzed the findings, providing evidence for the effectiveness of WS in treating neurodegenerative diseases such as Alzheimer’s, Huntington’s, and Parkinson’s (17, 39–41). One of the primary reasons for utilizing ashwagandha products is to alleviate stress, which is a common practice in some populations. There is widespread acknowledgement that stress can lead to both physiological and anatomical alterations within the brain, and stress has been linked to the development of several neuropsychiatric conditions such as anxiety, depression, and insomnia (42). The ways in which stress plays a role in these disorders involve increased activity of the hypothalamic–pituitary–adrenal (HPA) axis and disruption of the normal function of the immune system (43, 44). Based on the strong connection between stress and neuropsychiatric disorders, the anti-stress properties of WS are believed to play a crucial role in its potential benefits for depression, anxiety, and insomnia.

The mechanism of anxiety largely involves GABAergic neurotransmission, as GABA is the primary inhibitory neurotransmitter in the central nervous system (45). The chief location where GABA agonist drugs exert their effects is at GABA type A (GABAA) receptors, which enhance GABAergic function, and these drugs are frequently prescribed for managing anxiety disorders (46). Extensive research in non-human subjects has indicated that substances present in WS actively engage and regulate GABAA receptors, potentially explaining the anxiety-reducing effect of WS. The initial evidence of the ability of WS to mimic GABA was reported by Mehta et al. in 1991. The researchers discovered that a methanolic extract of WS root increased the influx of chloride ions in spinal cord neurons of mammals without the presence of GABA. The extract also hindered the binding of GABA to its receptor similar to how GABAA receptor agonists act (47). Research studies using receptor-binding assays have confirmed that the substances found in methanolic extracts of WS root show a strong binding to GABAA receptors, while they show a noticeably weaker binding to GABAB, glutamatergic, and opioid receptors (48). The GABAA receptor-specific function of WS has been confirmed by various animal experiments. The effects of morphine and ethanol on the dopamine-producing neurons in the ventral tegmental area of the rat brain were inhibited by a methanolic extract of WS roots. This was attributed to the activation of the GABAA pathway, rather than the GABAB pathway (49). In a mouse experiment, it was observed that a small amount of an unspecified extract from WS roots helped increase the threshold for seizures caused by pentylenetetrazol (PTZ). This effect was enhanced when combined with low doses of GABA to activate GABA receptors, and diazepam, which alters the function of GABAA receptors (50). In a previous mouse experiment, Kulkarni and colleagues (1993) reported that a methanolic extract of WS (specific plant parts not mentioned) in conjunction with pentobarbital (a GABAA receptor activator) resulted in better defense against PTZ-induced damage, compared to each agent on its own (51). Moreover, when a lower dose of WS was added to GABA, it enhanced the protective properties of the extract.

In a model using rats to simulate sleep disruption, the administration of a specific GABAA activator (muscimol) increased the sedative properties of an unspecified root extract from the WS plant. Conversely, the use of a GABAA receptor blocker (picrotoxin) counteracted this effect (52). Inhibitors of GABAA receptors, specifically picrotoxin and bicuculline, completely abrogated the ability of both methanolic and aqueous extracts from WS roots to cause depolarization in gonadotropin releasing hormone neurons in mice. These inhibitors also prevented the increase of inward ion currents in substantia gelatinosa neurons in mice, as well as in GABAA channels in rat brains (53).

**Figure 3:**
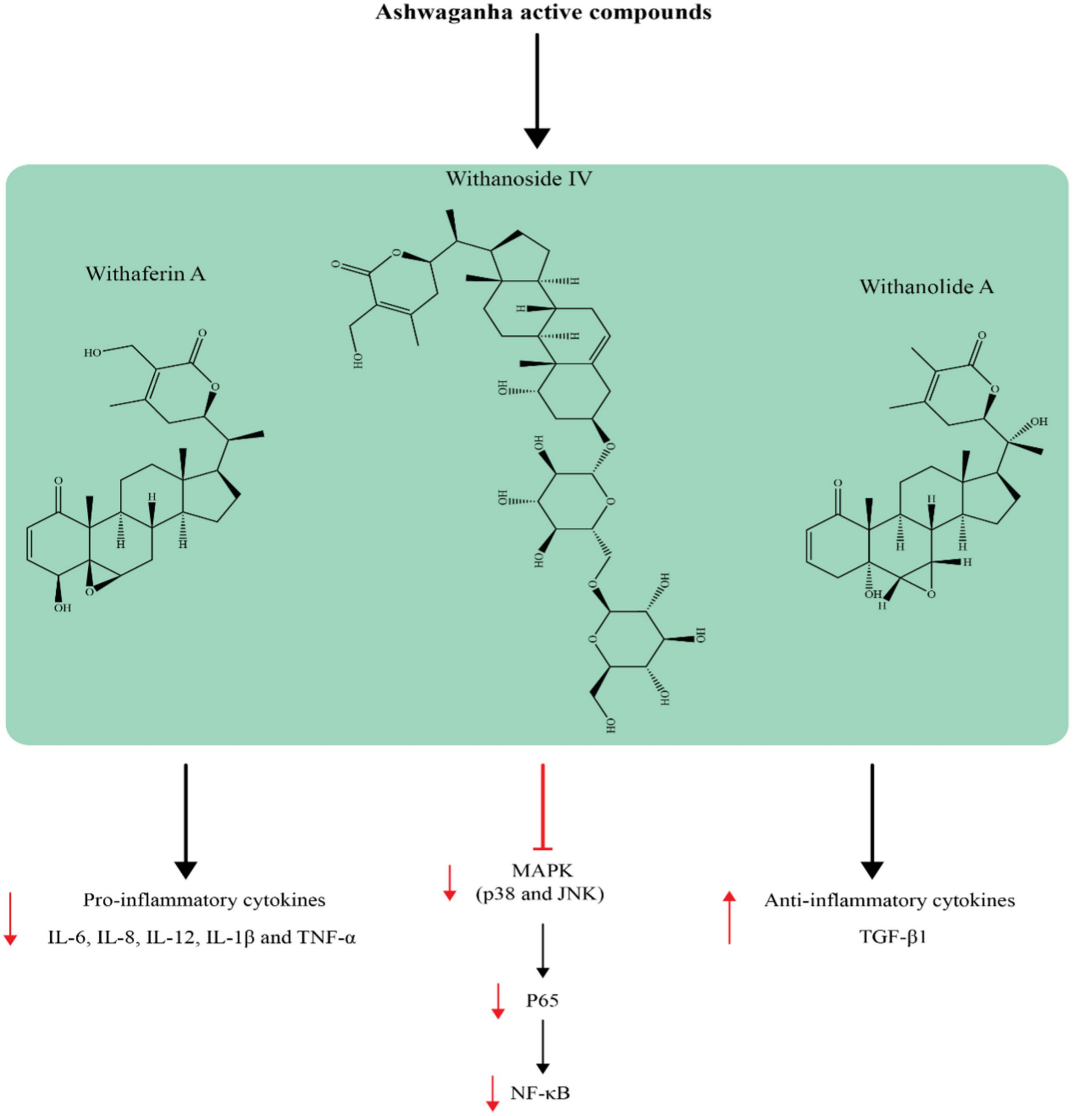
The effects of ashwagandha on inflammatory markers.

Because the brain is particularly vulnerable to oxidative stress, and anxiety disorders are characterized by a decrease in protective antioxidants as well as an increase in oxidative damage, treatments that protect against oxidative stress are desirable (54, 55). Multiple suggested mechanisms have been suggested to explain how oxidative stress plays a role in brain disorders. Oxidative stress can act as a trigger for these disorders and can also be a result of neuroinflammation, which has also been linked to brain-related disorders (56). Figure 3 shows how the components of ashwagandha can affect cell signaling pathways and the production of inflammatory mediators. Inflammation in peripheral tissues may directly play a role in increasing neuroinflammation and oxidative stress in the brain (56). Although there is evidence linking both oxidative stress and inflammation to brain-related disorders, it has not been conclusively proven which one causes the other or vice versa (57). Studies on animals exploring the effects of WS on anxiety have revealed a strong link between improvements in anxiety-related behavior and the amelioration of oxidative stress and inflammatory indicators.

The root extract and leaf extract of WS, although not clearly described, were able to increase the levels of catalase activity and reduced glutathione (GSH) in the brain, and also lower the levels of lipid peroxidation in a mouse model of sleep deprivation and a zebrafish model of neurotoxicity induced by benzo[a]pyrene (58, 59). Furthermore, WS demonstrated the ability to decrease nitrite levels in the mouse model and also lower protein carbonylation in the zebrafish model (58, 59). In an experiment using rats to simulate an ischemic stroke, a uniform hydroalcoholic extract derived from WS roots effectively decreased the levels of lipid peroxidation and enhanced antioxidant function in the brain (60). ASH-WEX, an aqueous leaf extract, was shown to decrease pro-inflammatory cytokines, specifically TNFα and IL-6, both in the peripheral and central nervous systems, in animal models of neuroinflammation and sleep deprivation (61). ASH-WEX produced a significant reduction in indicators of reactive gliosis, such as GFAP, as well as neuroinflammation, such as NOX2, iNOS, and COX2. Furthermore, it successfully regulated various inflammatory pathways and reduced cellular death in the brain (62). In a rat experiment examining the effects of a high fat diet on obesity, the use of WS dry leaf powder significantly lessened the expression of pro-inflammatory cytokines both in the body and brain, decreased indicators of reactive gliosis and neuroinflammation, modulated the nuclear factor NF-kappa-B (NF-κB) pathway, and lowered cell death (63).

Supplementation with ashwagandha has been shown to lower C-reactive protein (CRP) activity (64), one of the most important indicators of inflammation. Furthermore, other studies have shown that ashwagandha exerts a number of anti-inflammatory effects on chronic inflammation-mediated diseases (65), particularly rheumatoid arthritis (66), inflammatory bowel disorder (67), and systemic lupus erythematosus (68). These studies suggest that ashwagandha may be a useful tool for reducing the cytokine storm. Withanolides, particularly Withaferin A, are responsible for the majority of ashwagandha’s anti-inflammatory effects, according to several reports (69, 70). Ashwagandha may work by interacting with components of the proinflammatory cell signaling pathway, such as NF-κB, signaling kinases, HSP90, Nrf2, and the inflammasome complex, even though the mechanisms behind the anti-inflammatory effect of withanolides are not fully understood (71). Since the NF-κB transcription factor family is implicated in a number of chronic disorders caused by inflammation, individuals with high NF-κB levels may benefit from therapeutic targeting of NF-κB. In this situation, ashwagandha has the ability to inhibit and mediate the activity of the NF-κB pathway (72). It is thought that strong protein kinase inhibitor activity is necessary for ashwagandha to function. Ashwagandha has the ability to inhibit the signaling cascades of protein kinases, which are essential in inflammatory pathways (72). Furthermore, it appears that kinase inhibition takes place when nitric oxide synthesis is inhibited, which also benefits the inflammatory process. Another possible explanation for ashwagandha’s anti-inflammatory properties is the downregulation or destabilization of HSP activity, which is implicated in regulatory kinase pathways. As previously mentioned, ashwagandha regulates Nrf2 to moderate oxidative stress (34, 73). Nrf2 activation may account for ashwagandha’s anti-inflammatory properties, as oxidative stress frequently takes place in sites of inflammation and is thought to be one cause of chronic inflammation (74). Finally, by blocking inflammasomes, cytokines, and other multiprotein pro-inflammatory complexes, ashwagandha may lessen inflammation (72, 75).

According to some reports, ashwagandha is an adaptogen that, due to its antioxidant properties, boosts immunity, helps the body respond to stress more effectively, increases resilience, and fights oxidative stress and cellular damage (76, 77). It has been demonstrated that the bioactive C28-steroidal lactones present in WS leaves possess neuroprotective, anxiolytic, antioxidant, and anti-inflammatory properties. Moreover, Withanoside IV and its metabolite sominone, found in WS roots, have been shown to promote synaptogenesis and neuronal outgrowth. Furthermore, it has been demonstrated that the herb inhibits acetylcholinesterase and protects rats from cognitive decline (78). One study evaluated the effects of ashwagandha root extract 300 mg twice a day in humans with moderate cognitive impairment (79). After 8 weeks, the treatment group outperformed the placebo group in tests measuring immediate and general memory, information-processing speed, executive function, and attention. However, the benefits on working memory and visuospatial processing were not definitive because there was little difference between the two groups’ performance on these tasks. Another study examined the effect of ashwagandha extract on cognitive impairment in individuals with bipolar disorder (80). Tests were conducted at baseline and after the intervention, with rats randomly allocated to receive 500 mg/day of ashwagandha or a placebo for 8 weeks. When compared to the placebo, subjects in the treatment group showed significantly better results on the Flanker Test (neutral mean reaction time), the Penn Emotional Acuity Test (mean social cognition response rating), and the Auditory Digit Span (mean digit span backward). These findings suggested that ashwagandha extract may safely enhance cognitive function in bipolar disorder patients, including verbal working memory, response time, and social cognition response. Another study was carried out using ashwagandha root extract on a group of horses. The animals were subjected to a variety of stressors, including loud noises, prolonged physical activity, and separation. Following a 21-day period, the treated group showed a statistically significant reduction in cortisol, glucose, adrenaline, IL-6, lipids, creatinine, aspartate aminotransferase, and alanine aminotransferase (81).

## Effects of ashwagandha on sports performance

The physiological measure known as maximum oxygen consumption, or VO2 max, acts as a measure of an individual’s aerobic capability. It is a measure of cardiorespiratory fitness that governs both athletic performance and overall health condition (82, 83). With an emphasis on competitive sports, the VO2 max is one of the important variables that control success in endurance activities (84), along with running efficiency and the anaerobic threshold. It also helps to improve team sports performance by raising the intensity of work, the distance covered, and the quantity of sprints completed (85, 86). In the realm of general health, VO2 max holds particular significance beyond athletic performance. In adults and the elderly, low VO2 max values have been linked to an elevated risk of death and the loss of an independent lifestyle (87), whereas high cardiorespiratory fitness levels have been linked to a lower risk of cardiovascular disease (88, 89). Since a stronger aerobic capacity is linked to a higher quality of life, the VO2 max level is also significant in children (90).

Table 1 lists some clinical trials that have examined the effect of ashwagandha on VO2 max, sports performance and metabolic profiles. The ability of Indian cyclists to sustain cardiorespiratory endurance was assessed in a clinical trial of ashwagandha supplements. Forty top-level Indian cyclists were divided into groups for the intervention and placebo. For 8 weeks, the experimental group was given 500 mg capsules containing aqueous ashwagandha root extract twice a day, while the placebo group received starch capsules for 8 weeks. Compared to the placebo group, which did not exhibit any changes in baseline levels, the experimental group showed a substantial improvement in all parameters, including VO2 max, time to exhaustion on the treadmill, and METS (metabolic equivalents) (91). The conclusions showed that the anaerobic capacity of both males and females had significantly improved (92).

**Table 1:** Clinical trials examining the effects of ashwagandha on VO2 max, sports performance and metabolic profiles.

Participants	Dosage	Duration	Results	Reference
Elite athletes	1,000 mg/day	8 weeks	Improved the cardiorespiratory endurance	(91)
Elite athletes	1,000 mg/day	8 weeks	Improved the anaerobic capacity	(92)
Healthy young men engaged in resistance training	600 mg/day	8 weeks	Significant increases in strength and muscle mass	(93)
Healthy young adults	500 mg/day	8 weeks	Increased velocity, power and VO2 max	(94)
Healthy athletic adults	600 mg/day	8 weeks	Enhanced cardiorespiratory endurance Improved the quality of life	(26)
Young hockey players	500 mg/day	8 weeks	Improved agility levels	(95)
Athletic adults	600 mg/day	12 weeks	Enhanced the cardiorespiratory endurance Improved quality of life	(27)
Hockey Players	1,000 mg/day	8 weeks	Improved VO2 max and hemoglobin concentrations	(96)
Hockey Players	1,000 mg/day	8 weeks	Improved Core Muscle Strength and Stability	(97)
Healthy participants	600 mg/day	8 weeks	Improved muscle strength, growth and endurance	(98)
Male sprinters	2.5–3 g/day	12 weeks	Improved standing broad jump, 50-yard dash, pull-ups, sit-ups, and shuttle run	(99)
Elite cyclists	1,000 mg/day	8 weeks	Improved the aerobic capacity	(100)
Healthy subjects	12 g/day	60 days	Improved hemoglobin and VO2 max	(101)
Healthy subjects	500 mg/day	12 weeks	Improved upper and lower-body strength	(102)
Healthy subjects	20 g/day	4 weeks	Improved Harvard Step Score and VO_2_ max.	(103)

Another study was carried out on healthy subjects undergoing resistance training to investigate the potential benefits of ashwagandha root extract intake on strength and muscle mass. Fifty-seven young male individuals with no prior resistance training experience were divided into treatment and placebo groups for the 8-week trial. The control group was given starch placebo capsules, while the treatment group received 300 mg/twice a day of ashwagandha root extract. After completing baseline assessment, both subject groups engaged in resistance training for 8 weeks, with follow-up measures conducted at the conclusion of the eighth week. The 1-RM load for the bench press and leg extension movements was used to assess muscle strength. Creatine kinase levels, a measure of muscle damage caused by exercise, was used to assess muscle healing. When compared to the group of subjects given a placebo, those treated with ashwagandha saw a significant increase in muscle strength when performing the bench-press exercise (an increase of 46.0 kg compared to 26.4 kg for the placebo group), as well as the leg-extension exercise (an increase of 14.5 kg compared to 9.8 kg for the placebo group). Additionally, the ashwagandha group also had a greater increase in muscle size at the arms (5.3 cm^2^ compared to 7.2 cm^2^ for the placebo group). These results were statistically significant, with *p*-values of 0.001 and 0.04 for the bench-press and leg-extension exercises, respectively. According to the study, participants who took ashwagandha had significantly improved muscle strength in their arms (8.6 cm, 95% CI, 6.9, 10.8; *p* = 0.01) and chest (3.3 cm, 95% CI, 2.6, 4.1; *p* < 0.001) compared to those who took the placebo. Additionally, those taking ashwagandha also showed decreased levels of exercise-induced muscle damage, as shown by their stabilized serum creatine kinase levels (1307.5 U/L, 95% CI, 1202.8, 1412.1) compared to the placebo group (1.4 cm, 95% CI, 0.8, 2.0). Ashwagandha supplementation resulted in a significant increase in catalase activity (1462.6 U/L, 95% CI, 1366.2, 1559.1; *p* = 0.03), a significantly higher rise in testosterone levels (18.0 ng/dL, 95% CI, −15.8, 51.8 vs. 96.2 ng/dL, 95% CI, 54.7, 137.5; *p* = 0.004), and a significant decrease in body fat percentage (1.5, 95% CI, 0.4, 2.6% vs. 3.5, 95% CI, 2.0, 4.9%; *p* = 0.03) compared to the placebo group (93).

Ashwagandha and arjuna (*Terminalia arjuna*) were shown to have different effects on the average absolute and relative power, VO2 max, maximum velocity, blood pressure, and balance in humans, both separately and in combination. Forty healthy subjects were assigned to experimental groups, 10 of whom received standardized extracts of ashwagandha root, 10 of whom received standardized extracts of *Terminalia arjuna* bark, 10 of whom received a combination of both herbal extracts, and the remaining 10 received flour-filled capsules as a placebo. Each medication was administered as capsules, with a daily dose of 500 mg. Ashwagandha was shown to improve power, velocity, and VO2 max, whereas arjuna was found to boost VO2 max and decrease resting systolic blood pressure. All indicators displayed improvement when both extracts were administered together, with the exception of diastolic blood pressure and balance (94).

Another study evaluated the effectiveness and safety of ashwagandha in improving cardiorespiratory endurance in fit subjects. Fifty healthy, fit individuals were randomly divided into two groups and given equal amounts of placebo and ashwagandha for 8 weeks. The ashwagandha group was given 300 mg of ashwagandha root extract capsules, twice a day. VO2 max testing and cardiorespiratory endurance was evaluated. The findings showed that the ashwagandha group’s VO2 max value was significantly higher than the placebo group. By the conclusion of the trial, the participants in the ashwagandha group showed a statistically significant rise above their baseline. Those in the ashwagandha group showed significantly higher Total Quality Recovery Scores (TQR) than those in the placebo group. When comparing the Ashwagandha group to the placebo group, the Daily Analysis of Life Demands for Athletes (DALDA) questionnaire revealed statistical significance. Better results were also obtained in the Recovery-Stress Questionnaire for Athletes (RESTQ) evaluation, particularly for lack of energy, fatigue recovery, and fitness analysis. In the ashwagandha group, there was also a notable increase in antioxidant levels (26).

An investigation into the effect of ashwagandha supplementation on hockey players’ agility level was carried out. A total of 52 male hockey players were randomly separated into two groups. Group II was a placebo control group and Group I was the experimental group receiving WS. For 8 weeks, the experimental group took ashwagandha 500 mg/twice a day, whereas the placebo group took starch capsules. The Illinois Agility Run Test Getchell Test was used to measure the level of agility in the control and experimental groups prior to and following the consumption of placebo or ashwagandha. The results showed that after 4 and 8 weeks, the agility level in the ashwagandha group had significantly improved. However, there was no discernible improvement in agility levels in the placebo group (95). Another report investigated the effects of ashwagandha supplements on hockey players’ VO2 max and hemoglobin levels (Hb) in this group of hockey players. Before and after the ashwagandha and placebo were administered, the experimental and control group VO2 max and Hb were monitored. The conclusions showed that the experimental group’s VO2 max and Hb had significantly improved (96).

After 4 and 8 weeks, respectively, the experimental group core muscle strength and stability had significantly improved. On the other hand, following 8 weeks of placebo treatment, there was no discernible increase in core muscle strength & stability in the control group (97).

In a different trial, 50 healthy athletes were assessed for their quality of life and how well their cardiorespiratory endurance was improved by ashwagandha root extract. A 20-meter shuttle run test was used to measure VO2 max in order to evaluate cardiorespiratory endurance. The findings showed that after 8 and 12 weeks, ashwagandha supplementation increased mean VO2 max from baseline more than the placebo. At 12 weeks, the quality of life scores in the ashwagandha group considerably improved in comparison to the placebo group in all subdomains (27).

An investigation was conducted on the effects of 600 mg of standardized ashwagandha on strength, muscle mass, and cardiorespiratory endurance after resistance exercise. Eighty healthy male and female volunteers who regularly exercised were divided into two groups and given either 300 mg of ashwagandha supplements twice a day for 8 weeks, or an equivalent placebo. Every participant engaged in resistance training for 8 weeks. VO2 max, muscle mass, and strength were measured at baseline and after 8 weeks. According to the findings, ashwagandha improved endurance, leg press, and bench press performance more than a placebo. Moreover, both male and female ashwagandha users showed higher gains in muscle girth for the chest, arm, and thigh (98).

Another trial examined the impact of ashwagandha consumption on specific physical fitness metrics in male sprinters. The 20 male sprinters were split evenly between experimental and control groups. For 12 weeks, ashwagandha was administered to the experimental group three times a week on alternate days taken with milk. Following a 12-week period, the 20 male sprinters undertook a physical fitness test. According to the results, ashwagandha significantly improved the standing broad jump, number of pull-ups, 50-yard dash, number of sit-ups, and shuttle run performance (99).

Another study was designed to examine the effects of ashwagandha supplements on the physical performance of top Indian cyclists based on gender differences. The study used an identical experimental design with 13 males and 19 females totaling 38 cyclists. METs and VO2 max measures of aerobic capacity were assessed directly using the Bruce technique. For 8 weeks, the experimental group received ashwagandha supplements, whereas the control group received capsules containing starch. The findings showed that whereas average power and peak power greatly improved in females, VO2 max improved significantly in males. After 8 weeks, males using ashwagandha supplements showed improvements in their aerobic and anaerobic capacity (100).

The effectiveness of ashwagandha in increasing VO2 max in healthy participants was assessed in a controlled, single-blind, randomized clinical trial. Two groups each consisted of 54 healthy volunteers, the study group started the day with 12 g of ashwagandha Choorna and 200 mL of milk on an empty stomach, whereas the control group merely had 200 mL of milk. The VO2 max of the study and control groups was assessed using the Rockport fitness walking test on days zero, 60 and 90 of follow-up. It was discovered that the study group VO2 max and Hb had significantly improved (101).

The effect of Sensoril^®^ supplements on strength training adaptation was investigated in another trial. Men who undertook recreational activity were divided into double-blinded groups assigned to receive 500 mg/day of Sensoril^®^ or a placebo. Measurements included 7.5 km cycling time trial, clinical blood chemistry, DEXA measurements of muscle strength, endurance, power, and body composition, at baseline and after 12-weeks of supplementation. The subjects continued to eat normally and adhered to a resistance-training regimen of progressive overload (4 days per week, divided between upper and lower body). The results showed that S500 produced much higher increases in the 1-RM squat and bench press performance. A shift in the android/gynoid ratio determined by DEXA also favored S500. Systemic hemodynamics, visual analog scales for affect and recovery, and body composition did not show any between-group differences. However, only the S500 group showed significant improvements in peak bench press power, average squat power, 7.5 km time trial performance, and perceived recovery scores. There was only a small polycythemia effect in placebo, according to the clinical chemistry measurement, and no additional statistically or clinically significant alterations were found (102).

Rasayana dravyas is a potential dietary supplement containing granules of ashwagandha, Jeeraka (*Eleusine coracana*), Shatavari (*Asparagus racemosus*), Mudga (*Vigna radiata*), Ragi (finfer millet), and Shunti (*Zingiber officinale*). Twenty-three subjects participated in an open-label, double-arm, controlled clinical trial using a modified wait-listed crossover design. For the duration of the trial, they were given 150 mL of milk and 20 g of ashwagandhadi granules in Rasayana dravyas daily for 1 month. The results revealed a statistically significant difference in the VO_2_ max and Harvard Step Score for the treatment group compared to the placebo group (103).

### Practical applications

This review has provided useful information that will help sports performance practitioners in real-world scenarios, and to understand how supplements designed to reduce and control ROS damage work. In particular, it focuses on ashwagandha supplementation for improving athletic performance and modulating antioxidant response. In order to boost performance, sports teams may decide to adopt new dietary guidelines and working practices as a result of this evaluation.

## Conclusion

Evidence suggests that ROS are key players in physiological signaling networks that control how the body reacts to exercise, and that physical activity-induced oxidative stress and inflammation are necessary for training adaption. The process of physiological adaptation requires a proper balance between antioxidants and free radicals, and it is possible that athletes who take antioxidants could counteract the advantageous effects of ROS in normal cell signaling. According to this theory, ROS produced during exercise are thought to activate PGC1-α and MAPK, two biochemical pathways essential for both muscle growth and aerobic capacity. Some studies have proposed that a brief rise in reactive oxygen species (ROS) brought on by physical activity may have advantageous consequences, including controlling the contraction of muscles, promoting muscle regeneration, and enhancing vasodilation while exercising. On the other hand, oxidative stress and elevated ROS levels can damage tissues and cells and induce inflammation. Many endurance athletes consume diets deficient in antioxidants to maintain their high demands for energy to carry out physical activity, despite their demanding training schedules. Although ashwagandha pills are frequently advised for endurance athletes, the numerous health advantages of exercise alone may make then unnecessary.

## Author contributions

SG: Data curation, Investigation, Methodology, Project administration, Resources, Software, Supervision, Visualization, Writing – original draft, Writing – review & editing. MR: Conceptualization, Data curation, Investigation, Methodology, Project administration, Supervision, Visualization, Writing – original draft, Writing – review & editing.
